# Supplementary material for: Variation in Road Dust Heavy Metal Concentration, Pollution, and Health Risk with Distance from the Factories in a City–Industry Integration Area, China
Source: Int J Environ Res Public Health. 2022 Nov 6;19(21):14562. doi: 10.3390/ijerph192114562 (PMC9656356; doi:10.3390/ijerph192114562)
Supplement: Supplementary file 1 [file ijerph-19-14562-s001.zip › ijerph-1961066-supplementary.pdf]

**Table S1.** Basic questionnaire of the chosen factories surroundings.

| Full name | Abbr. name | History/a | Area/km$^2$ | Products | Surrounding environment |
|---|---|---|---|---|---|
| The Foxconn | F1 | 8 | 0.11 | Electronic parts | N-Industrial and residential areas; S-The square and logistics center; W-Various factories; E-Railway and highway intersection zone |
| Zhengzhou Nissan | F2 | 8 | 1.10 | Automobile and engine | N-Equipment manufacturing factory; S-Golf Course and park; W-Construction site E-Viaduct turntable |
| The Yutong group | F3 | 7 | 1.13 | large and medium bus | N-Village; S-Residence Community; W-Wasteland, auto repair factory; E-Farmland and wasteland |

**Table S2.** Exposure parameters used in the evaluation of health risks.

| Items | Symbol | Units | Value | References |
|---|---|---|---|---|
| Basic parameter | *c* | mg·kg$^{-1}$ /mg·m$^{-3}$ | 95%UCL | This study |
| Exposure behavior parameters | *ED* | a | 24 | [7,8] |
| | *BW* | kg | 58.6 | [24] |
| | *EF* | d/a | 350 | |
| | *AT*(non-cancer) | d | 365×ED | [25] |
| | *AT* (cancer) | | 365×71.4 | |
| Ingestion | *IngR* | mg·d$^{-1}$/m$^3$·d$^{-1}$ | 100 | [7,8] |
| Inhalation | *InhR* | m$^3$·d$^{-1}$ | 12.8 | |
| | *PEF* (soil and dust) | m$^3$·kg$^{-1}$ | 1.36×10$^9$ | |
| Dermal contact | *SA* | cm$^2$/ m$^2$·d$^{-1}$ | 2145 | [26] |
| | *SL* | mg·cm$^{-2}$ | 0.07 | [8] |
| | *CF* (soil and dust) | mg·kg$^{-1}$ | 1.0×10$^{-6}$ | [24,27] |
| | *ABS* | unitless | 0.03(As); 0.001 (for the other metals) | [27–29] |
| Non-carcinogenic reference dose | *RfD* | (kg·d)·mg$^{-1}$ | $RfD_{ingestion}$: 0.0003(As), 0.001 (Cd), | [27–29] |

| Items | Symbol | Units | Value | References |
| --- | --- | --- | --- | --- |
| | | | 0.003 (Co),<br>0.003 (Cr),<br>0.04 (Cu),<br>0.0003 (Hg),<br>0.047 (Mn),<br>0.02 (Ni),<br>0.0035 (Pb),<br>0.3 (Zn)<br>$RfD_{inhalation}$:<br>1.23E-04 (As),<br>1.00E-03 (Cd),<br>2.80E-05 (Co),<br>2.86E-05 ($Cr^{6+}$),<br>4.02E-02 (Cu),<br>3.00E-04 (Hg),<br>1.40E-05 (Mn),<br>2.06E-02 (Ni),<br>3.52E-03 (Pb),<br>3.00E-01 (Zn)<br>$RfD_{dermal}$:<br>3.00E-04 (As),<br>1.00E-05 (Cd),<br>6.00E-05 (Co),<br>6.00E-05 ($Cr^{6+}$),<br>1.20E-02 (Cu),<br>2.40E-05 (Hg),<br>2.40E-03 (Mn),<br>5.40E-03 (Ni),<br>5.25E-04 (Pb),<br>6.00E-02 (Zn) | |
| Carcinogenic slope factor | *SF* | (kg·d)·$mg^{-1}$ | $SF_{ingestion}$:<br>1.5 (As)<br>$SF_{inhalation}$:<br>15.1 (As),<br>6.4 (Cd),<br>9.8 (Co),<br>42 (Cr),<br>0.84 (Ni)<br>*Dermal* SF:<br>3.66 (As) | [28,29] |
